# Origin of the kink of somatic action potentials

**DOI:** 10.1186/1471-2202-16-S1-O8

**Published:** 2015-12-18

**Authors:** Maria T Teleńczuk, Marcel Stimberg, Romain Brette

**Affiliations:** 1 University Pierre et Marie Curie, Paris, 75006, France

## 

The Hodgkin and Huxley (1952) model of action potential (AP) generation accounts for many properties of APs observed experimentally and has been successfully used in modeling neurons of different types. In this model, however, the spike onset is much shallower than in experimental recordings from the soma suggesting different activation properties of sodium channels in the real tissue. To explain the origin of the observed sharpness (kink) in the spike onset three hypotheses were proposed: 1. Cooperative hypothesis: sodium channels cooperate in the axon initial segment, which makes their collective activation curve much sharper [1]. However, there is no experimental evidence for this hypothesis. 2. Active backpropagation hypothesis: spikes are initiated in the axon and backpropagate to the soma. The kink is caused by the sharpening of the axonal spike by active conductances during its backpropagation through the axon [2]. 3. Compartmentalization hypothesis: the kink comes from distal initiation and the current sink caused by the difference in the size of the soma and axon [3].

To find out what is truly happening in the cell during the action potential, we investigated the active backpropagation and compartmentalization hypotheses by means of computational modeling and theoretical analysis. In order to differentiate the hypotheses, we varied systematically the morphology of the neuron and distribution of the ionic channels along the cell, and tested how they contribute to the appearance of the kink. We show that the kink at spike onset is primarily due to compartmentalization rather than to active backpropagation.

**Figure 1:**
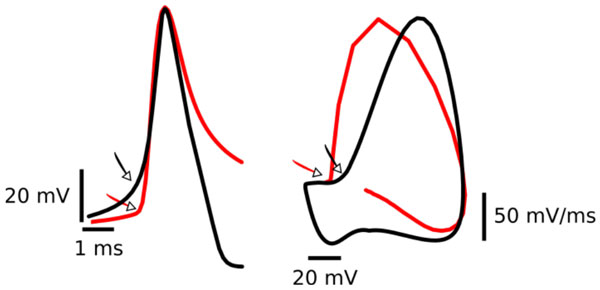
**Kink in the action potential**. Patch clamp recordings (red) from a cortical pyramidal cell and action potential produced by a Hodgkin Huxley type model (black). Left: Voltage-time relationship. Right: Phase plot of the same traces as in the left (dV/dt vs. V). Note, in both representations the onset of the action potential is much faster for the experimental recordings (red) than for the model (black).
